# New Role of JAK2/STAT3 Signaling in Endothelial Cell Oxidative Stress Injury and Protective Effect of Melatonin

**DOI:** 10.1371/journal.pone.0057941

**Published:** 2013-03-06

**Authors:** Weixun Duan, Yang Yang, Wei Yi, Juanjuan Yan, Zhenxin Liang, Ning Wang, Yue Li, Wensheng Chen, Shiqiang Yu, Zhenxiao Jin, Dinghua Yi

**Affiliations:** 1 Department of Cardiovascular Surgery, Xijing Hospital, The Fourth Military Medical University, Xi’an City, Shanxi Province, China; 2 Department of Medical Administration, Beidaihe Sanatorium, Beijing Military Area Command, Qinhuangdao City, Hebei Province, China; 3 Department of Prosthodontics, School of Stomatology, The Fourth Military Medical University, Xi’an City, Shanxi Province, China; UAE University, Faculty of Medicine & Health Sciences, United Arab Emirates

## Abstract

Previous studies have shown that the JAK2/STAT3 signaling pathway plays a regulatory role in cellular oxidative stress injury (OSI). In this study, we explored the role of the JAK2/STAT3 signaling pathway in hydrogen peroxide (H_2_O_2_)-induced OSI and the protective effect of melatonin against (H_2_O_2_)-induced injury in human umbilical vein endothelial cells (HUVECs). AG490 (a specific inhibitor of the JAK2/STAT3 signaling pathway) and JAK2 siRNA were used to manipulate JAK2/STAT3 activity, and the results showed that AG490 and JAK2 siRNA inhibited OSI and the levels of p-JAK2 and p-STAT3. HUVECs were then subjected to H_2_O_2_ in the absence or presence of melatonin, the main secretory product of the pineal gland. Melatonin conferred a protective effect against H_2_O_2_, which was evidenced by improvements in cell viability, adhesive ability and migratory ability, decreases in the apoptotic index and reactive oxygen species (ROS) production and several biochemical parameters in HUVECs. Immunofluorescence and Western blotting showed that H_2_O_2_ treatment increased the levels of p-JAK2, p-STAT3, Cytochrome c, Bax and Caspase3 and decreased the levels of Bcl2, whereas melatonin treatment partially reversed these effects. We, for the first time, demonstrate that the inhibition of the JAK2/STAT3 signaling pathway results in a protective effect against endothelial OSI. The protective effects of melatonin against OSI, at least partially, depend upon JAK2/STAT3 inhibition.

## Introduction

Endothelial cells are crucial for maintaining the physiological functions of the cardiovascular system [1]. Increasing evidence suggests that oxidative stress in endothelial cells, as characterized by an imbalanced cellular capability to produce and eliminate reactive oxygen species (ROS), is involved in the pathophysiology of several vascular diseases, such as atherosclerosis, diabetes and hypertension [2]. Hydrogen peroxide (H_2_O_2_) is widely used to mimic oxidative stress-induced injury within a short time period [3]. Although multiple cytokines and signaling pathways have been implicated in oxidative stress-mediated vascular damage [4], [5], the underlying pathophysiological mechanisms of oxidative stress injury (OSI) have not been fully elucidated.

The Janus kinase/signal transducer and activator of transcription (JAK/STAT) pathway is the signaling target of such pro-inflammatory cytokines as IL-6, which plays an important role in OSI [6]. Thus far, four mammalian JAKs (JAK1, 2, 3 and Tyk2) and seven mammalian STATs (STAT1, 2, 3, 4, 5a, 5b and 6) have been identified [7]. The JAK2/STAT3 signaling pathway is a highly evolutionarily conserved pathway that is involved in growth and development and controls communication among cells, signaling transduction in the cytoplasm and gene transcription in the nucleus [8]. JAK2/STAT3 signaling also affects cellular activities, such as proliferation, migration, growth, differentiation and death [9]. In recent years, many studies have confirmed that the JAK2/STAT3 signal pathway is hyper-activated in cellular and animal models of OSI, suggesting an important role of this signaling pathway in regulating oxidative stress responses [10], [11]. Indeed, it has been verified that H_2_O_2_-induced cell apoptosis and death are directly dependent on JAK2 and STAT3 activation [12], [13]. Accordingly, the modulation of the JAK2/STAT3 signaling pathway may provide an effective therapeutic strategy in the treatment of OSI.

Melatonin (N-acetyl-5-methoxytryptamine), the main secretary product of the pineal gland, is potentially effective in the prevention of a number of diseases involving free radical processes and has a wide spectrum of biological functions [14], such as cardioprotection [15], anti-inflammatory [16], antioxidant [17] and anti-cancer [18] properties, without toxic and mutagenic activities [19]. Melatonin has been tested as a potential therapeutic agent in a number of pathological conditions, including cardiovascular disease and other vascular dysfunctions [20], [21], and recent reports indicated that melatonin attenuated OSI in multiple organs under various pathological conditions [22]–[26]. In addition, the JAK2/STAT3 signaling pathway plays an important role in the biologic effects of melatonin [8], [15], [27]–[29]. However, whether JAK2/STAT3 signaling is involved in the protective effect and mechanism of melatonin against H_2_O_2_-induced OSI has not been studied to date.

In this study, we explored the role of the JAK2/STAT3 signaling pathway in H_2_O_2_-induced OSI in human umbilical vein endothelial cells (HUVECs). We then investigated whether melatonin protected the HUVECs from H_2_O_2_-induced injury via inhibition of the JAK2/STAT3 signaling pathway.

## Materials and Methods

### Materials

AG490, melatonin, 4′,6-diamino-2-phenylindole (DAPI), MTT [3-(4,5-dimethylthiazol- 2-yl)-2,5-diphenyltetrazolium bromide] and 2′,7′-dichlorofluorescein diacetate (DCFH-DA) were purchased from Sigma-Aldrich (St. Louis, MO, USA). Antibodies against JAK2 siRNA, Bax, Cytochrome c, p-JAK2, t-JAK2, p-STAT3 and p-STAT3 were purchased from Santa Cruz Company (Santa Cruz, CA, USA). Terminal deoxynucleotidyl transferase dUTP nick end-labeling (TUNEL) kits were purchased from Roche Company (Mannheim, Germany). The kits for the measurement of the lactate dehydrogenase (LDH), methane dicarboxylic aldehyde (MDA), superoxide dehydrogenase (SOD) and glutathione peroxidase (GSH-Px) concentrations were purchased from Institute of Jiancheng Bioengineering (Nanjing, Jiangsu, China). Anti-Bcl2, -Cytochrome c, -Caspase3 and -GAPDH antibodies were purchased from Cell Signaling Company (Boston, MA, USA). The rabbit anti-goat, goat anti-rabbit and goat anti-mouse secondary antibodies were purchased from Zhongshan Company (Beijing, China).

### Cell Culture and Treatments

HUVECs (ATCC CRL-1730; Shanghai Tiancheng Technology Company, China) were cultured in RPMI 1640 medium (Hyclone, UT, USA) supplemented with fetal calf serum (10%), 2 mM L-glutamine, 100 U/ml penicillin and 100 g/ml streptomycin at 37°C in 5% CO_2_ and 95% air. The melatonin stock solution was prepared in dimethylsulfoxide (DMSO) and diluted with culture medium immediately prior to use; 0.01% DMSO was used as a sham control. The cells were treated with H_2_O_2_ (400 µM) in the absence or presence of melatonin, AG490 and JAK2 siRNA for different intervals. The cells were harvested after the treatments for further analysis.

### Cell Viability Analysis

Cell viability was measured using the MTT assay. Briefly, after the cells were treated and washed with PBS, 10 µl of MTT dye was added to each well at a final concentration of 0.5 mg/ml. After 4 h of incubation, 100 µl of DMSO, the solubilization/stop solution, was added to dissolve the formazan crystals, and the absorbance was measured using a microtiter plate reader (SpectraMax 190, Molecular Device, USA) at a wavelength of 490 nm. The cell viability was expressed as an optical density (OD) value. In addition, the cell morphology was observed under inverted/phase contrast microscopy, and images were obtained (Olympus BX61, Japan).

### Cellular Adhesion Ability Assay

The procedure was according to a previously described method [30], with minor modifications. In brief, after centrifugation and resuspension in basal medium with 5% fetal bovine serum, the treated HUVECs (1×10^4^ cell per well) were placed on fibronectin-coated 6-well plates and incubated for 30 min at 37°C. Gentle washing with PBS 3 times was performed after 30 min for adhesion. The adherent cells were stained with MTT and counted by independent blinded investigators. The number of adherent cells in the control group was set as 100%.

### Wound Healing Assay

As described previously [31], HUVECs were seeded in 6-well plates and were treated for different intervals. We subsequently scratched the confluent cell monolayers with a P200 pipette tip to produce three parallel “wounds” in each well, and then the cells were incubated with 5% fetal bovine serum for 8 h. The migrated cells were photographed using inverted/phase-contrast microscopy, and images were obtained (Olympus BX61, Japan). The mean distance between the two ends of each scratch was quantified by manual measurements. The control was set as 100%.

### Cellular Apoptosis Assay

Cellular apoptosis was analyzed with the TUNEL assay using an in situ cell death detection kit. According to the manufacturer’s instructions, a double-staining technique was used: after the HUVECs were fixed in paraformaldehyde (4%) for 24 h, TUNEL was performed to stain the apoptotic cell nuclei (green), and DAPI was used to stain all the nuclei (blue). The index of apoptosis was expressed as the number of positively stained apoptotic HUVECs/the total number of HUVECs counted×100%.

### Measurement of Intracellular Reactive Oxygen Species (ROS) Content

The measurement of the intracellular ROS was based on the ROS-mediated conversion of nonfluorescent 2′,7′-DCFH-DA into fluorescent DCFH, as described previously [32]. After the cells were seeded and treated in black 96-well plates, the cells were washed with PBS (pH 7.4) and then incubated with DCFH-DA (20 µM) in PBS at 37°C for 2 h. At the end of the incubation, the DCFH fluorescence of the cells in each well was measured at an emission wavelength of 530 nm and an excitation wavelength of 485 nm using an FLX 800 microplate fluorescence reader (Biotech Instruments Inc., USA). The background was cell-free conditions. The results were expressed as the percentage of the control group (100%) fluorescence intensity.

### LDH Release Measurement

LDH, an indicator of cell injury, was detected after the exposure to H_2_O_2_ with an assay kit according to the manufacturer’s protocol. The activity of enzyme was expressed as units per liter, and the absorbance was measured at 440 nm.

### Measurements of Intracellular SOD, GSH-Px and MDA Contents

As described previously [32], the activities of SOD, GSH-Px and MDA were all determined using commercially available kits, and all the procedures completely complied with the manufacturer’s instructions. The activities of the enzymes were expressed as units per milligram protein. The assay of the SOD activity was based on its ability to inhibit the oxidation of hydroxylamine by the O^2−^ produced from the xanthine-xanthine oxidase system. One unit of SOD activity was defined as the amount that reduced the absorbance at 550 nm by 50%. The assay for the GSH-Px activity was by quantifying the rate of oxidation of reduced GSH to oxidized GSH by H_2_O_2_ and catalyzed by GSH-Px. One unit of GSH-Px was defined as the amount that reduced the level of GSH at 412 nm by 1 µM in 1 min/mg protein. The MDA content was measured at a wavelength of 532 nm by reaction with thiobarbituric acid (TBA) to form a stable chromophore. The values of the MDA level were expressed as nanomoles per milligram protein.

### Small Interfering RNA (siRNA) Treatment

HUVECs were plated in 6-, 24- or 96-well plates and allowed to grow to sub-confluence. The cells were transiently transfected with a negative control siRNA or JAK2 siRNA using the Lipofectamine RNAiMAX reagent (Invitrogen, USA) in OPTI-MEM medium (Gibco, USA) for 48 h; the cells were then prepared for further experiments.

### Immunofluorescence Assay

After being fixed in paraformaldehyde (4%) for 15 min, the cells were permeabilized in 0.1% Triton X-100 for 10 min and blocked in 5% bovine serum albumin for 30 min at room temperature. The cells were then incubated with anti-JAK2 and anti-STAT3 goat polyclonal antibodies (1∶200) overnight at 4°C. Following washing with PBS, the cells were incubated with a rabbit anti-goat secondary antibody conjugated with TRITC (1∶200) for 2 h. The cells were then incubated with 3,3′-diaminobenzidine (0.02 mg/ml) for 2 min, washed with PBS and mounted wet using glycerol (50%, v/v). Images were obtained under a fluorescence microscope (BX51, Olympus, Japan) with a CCD camera (DP70, Olympus, Japan). The images were imported into Image Pro Plus 6.0 Software (Media Cybernetics Company, USA), and the pixels for each color were analyzed to represent the positively stained cells quantitatively; the result of the control group was defined as 100%.

### Western Blot Assay

Cells were homogenized in lysis buffer containing 50 mmol/L Tris–HCl (pH 7.3), 150 mmol/L NaCl, 5 mmol/L EDTA, 1 mmol/L dithiothreitol, 1% Triton X-100, and 1% protease inhibitor cocktail. The lysates were centrifuged (15 min at 12,000×g), and the resulting supernatant was transferred to new tubes and stored at −70°C. The protein concentrations were determined using the Bradford protein assay kit. The proteins were separated by SDS-PAGE electrophoresis and transferred to nitrocellulose membranes. The membranes were blocked for 1 h in Tris-buffered saline and Tween 20 (TBST, pH 7.6) containing 5% non-fat dry milk powder and thereafter incubated overnight at 4°C with antibodies against JAK2 and STAT3 (1∶500 dilution) and Bcl2, Cytochrome c, Caspase3, and GAPDH (1∶1000 dilution), followed by washing in TBST. The membranes were probed with different secondary antibodies (1∶5000 dilution) at room temperature for 90 min, followed by washing in TBST. The protein bands were detected using chemiluminescence and quantified with Quantity One software package (Bio-Rad Laboratories, UK); the results of the control group were defined as 100%.

### Statistical Analysis

All of the values are presented as the mean ± the standard error of the mean (SEM). Comparisons were performed using an ANOVA, and multiple comparisons were performed using post hoc least significant difference comparisons. A value of P<0.05 was considered to be statistically significant.

## Results

**Figure 1:**
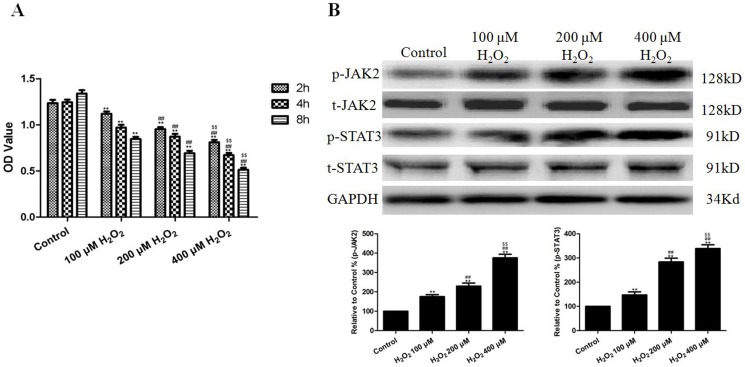
The effects of H_2_O_2_ on HUVEC viability and the levels of p-JAK2 and p-STAT3. (A) The viability of the HUVECs was assessed by performing an MTT assay, and the viability was expressed as an OD value. (B) Representative images of the Western blots are shown (treated for 4 h). The results are expressed as the mean ± SEM, n = 6, **P<0.01 compared to the control group, ^##^P<0.01 compared to the 100 µM H_2_O_2_ group, ^$$^P<0.01 compared to the 200 µM H_2_O_2_ group. OD, optical density.

HUVECs were subjected to 2, 4, and 8 h of H_2_O_2_ (100, 200, and 400 µM) treatment. As expected, incubation with H_2_O_2_ at different concentrations caused a significant decrease in the OD value (P<0.01, compared to the respective control groups), and the viability of the HUVECs was reduced by H_2_O_2_ in dose- and time-dependent manners (Figure 1A and Table S1). The results of Western blotting suggest that 4 h of H_2_O_2_ (100, 200, and 400 µM) treatment significantly increased the levels of p-JAK2 and p-STAT3 in a dose-dependent manner (Figure 1B). In addition, we carried out an additional experiment to find a lower concentration of H_2_O_2_ which had no effect on the cell viability. The results indicated that the concentration of H_2_O_2_ which was lower than 50 µM (treated for 4 h) had no effect on the cell viability. However, the lower concentrations of H_2_O_2_ (50 and 25 µM) also slightly up-regulated p-JAK2 and p-STAT3 (Figure S1A, S1B and Table S2).

**Figure 2:**
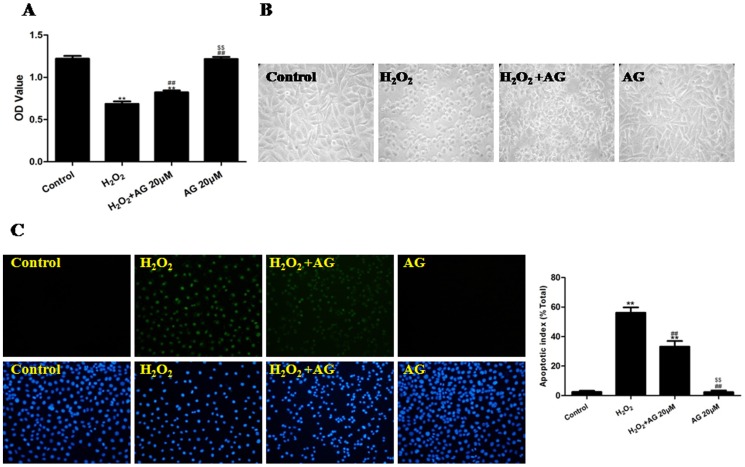
The effects of AG490 on the viability, morphology and apoptotic rate of H_2_O_2_-injured HUVECs (treated for 4 h). (A) The viability of the HUVECs was assessed by performing an MTT assay, and the viability was expressed as an OD value. (B) The cell morphology was observed using inverted/phase-contrast microscopy, and images were obtained. Significant cell shrinkage and a decreased cellular attachment rate was observed in the H_2_O_2_ group, yet combined treatment with AG490 reduced H_2_O_2_-induced cell shrinkage and decrease in the cellular attachment rate. (C) The apoptosis of the HUVECs was assessed by performing a TUNEL assay, and cell apoptosis was expressed as the apoptotic index. TUNEL staining was performed to stain the nuclei of the apoptotic cells (green), and DAPI was used to stain all of the nuclei (blue). The apoptotic index was expressed as the number of positively stained apoptotic cells/the total number of cells counted ×100%. The results are expressed as the mean ± SEM, n = 6, **P<0.01 compared to the control group, ^##^P<0.01 compared to the H_2_O_2_ group, ^$$^P<0.01 compared to the H_2_O_2_+ AG (20 µM) group. AG, AG490; OD, optical density.

To explore the role of the JAK2/STAT3 signaling pathway in the H_2_O_2_-induced OSI of HUVECs, the cells were subjected to 4 h of H_2_O_2_-induced OSI in the absence or presence of AG490 (20 µM). The H_2_O_2_ treatment significantly decreased the cell viability (P<0.01, compared with the control group). As observed using microscopy, the H_2_O_2_ treatment resulted in significant cell shrinkage and a decrease in the rate of cellular attachment compared to the control group. The AG490 treatment significantly increased cell viability (P<0.01, compared to the H_2_O_2_ group), attenuated H_2_O_2_-induced cell shrinkage and improved the attachment rate of the cells. Compared to the control group, the treatment with AG490 alone had no effect on cell viability (Figure 2A, 2B and Table S3). In addition, H_2_O_2_ treatment increased the cellular apoptotic index, whereas AG490 treatment significantly decreased the cell apoptotic index (P<0.01, compared to the H_2_O_2_ group, Figure 2C).

**Figure 3:**
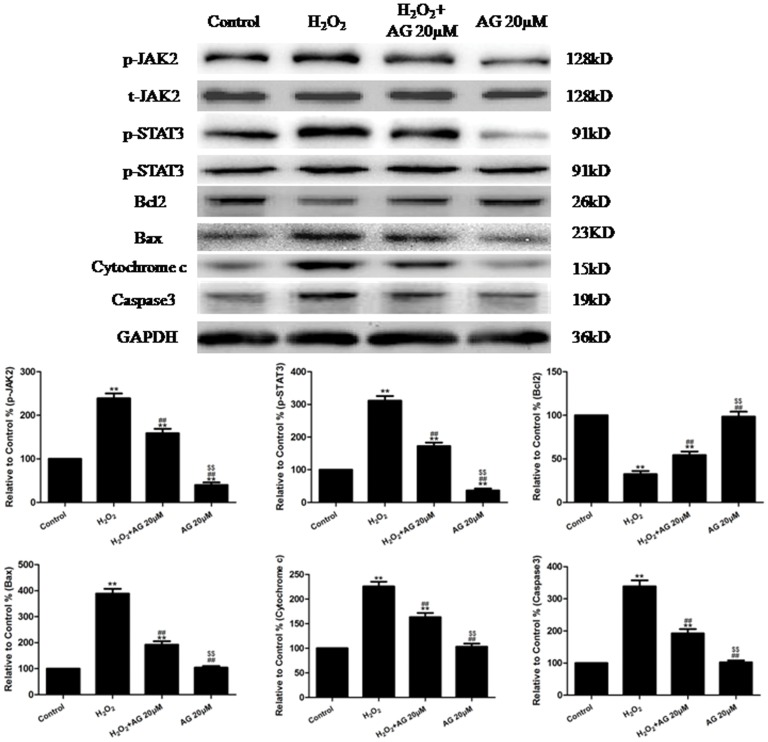
The effects of AG490 on p-JAK2 and p-STAT3 levels and the expression of Bcl2, Caspase3, Bax and cytochrome c in H_2_O_2_-injured HUVECs (treated for 4 h). Representative images of the Western blots are shown. The results are expressed as the mean ± SEM, n = 6, **P<0.01 compared to the control group, ^##^P<0.01 compared to the H_2_O_2_ group, ^$$^P<0.01 compared to the H_2_O_2_+ AG (20 µM) group. AG, AG490.

As depicted in Figure 3, our Western blotting analysis suggested that the H_2_O_2_ treatment significantly increased the levels of p-JAK2 and p-STAT3 and the expression of Caspase3, Bax and Cytochrome c compared to the control group (P<0.01); conversely, the treatment with H_2_O_2_ produced a significant decrease in the expression of Bcl2 (P<0.01, compared to the control group). However, treatment with H_2_O_2_+ AG490 (20 µM) produced a significant decrease in the levels of p-JAK2 and p-STAT3 and the expression of Caspase3, Bax and Cytochrome c (compared to the H_2_O_2_ group, P<0.01) and a significant increase in the expression of Bcl2 (compared to the H_2_O_2_ group, P<0.01). An immunofluorescence assay was also used to detect the expression of p-JAK2 and p-STAT3. As depicted in Figure S2A and S2B, the H_2_O_2_ treatment produced a significant increase in the levels of p-JAK2 and p-STAT3 compared to the control group (P<0.01). In contrast, when the cells were treated with H_2_O_2_+ AG490 (20 µM), there was a significant decrease in p-JAK2 and p-STAT3 compared to the cells that were treated with H_2_O_2_ alone (P<0.01).

**Figure 4:**
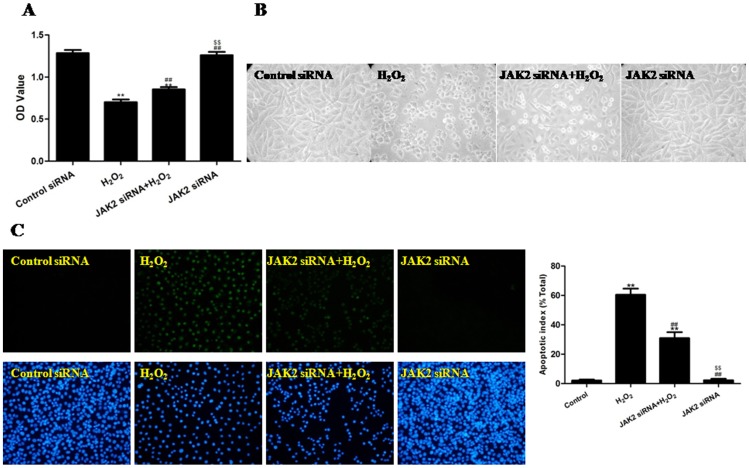
The effects of JAK2 siRNA on the viability, morphology and apoptotic rate of H_2_O_2_-injured HUVECs (treated for 4 h). (A) The viability of the HUVECs was assessed by performing an MTT assay, and the viability was expressed as an OD value. (B) The cell morphology was observed using inverted/phase-contrast microscopy, and images were obtained. Significant cell shrinkage and a decrease in the cellular attachment rate was observed in the H_2_O_2_ group, whereas the pretreatment with JAK2 siRNA reduced H_2_O_2_-induced cell shrinkage and decreased the cellular attachment rate. (C) The apoptosis of the HUVECs was assessed by a TUNEL assay and was expressed as the apoptotic index. TUNEL staining was performed to stain the nuclei of the apoptotic cells (green), and DAPI was used to stain all of the nuclei (blue). The apoptotic index was expressed as the number of positively stained apoptotic cells/the total number of cells counted ×100%. The results are expressed as the mean ± SEM, n = 6, **P<0.01 compared to the Control siRNA group, ^##^P<0.01 compared to the H_2_O_2_ group, ^$$^P<0.01 compared to the JAK2 siRNA+H_2_O_2_ group. OD, optical density.

Because AG490 may affect multiple JAK/STAT signaling receptors in addition to JAK2/STAT3, it is necessary to confirm that the protective role against OSI conferred by AG490 was mediated by JAK2/STAT3 signaling. We used JAK2 siRNA to inhibit JAK2 specifically to determine whether the protective effect of AG490 could be replicated. The cells were pretreated with JAK2 siRNA and then subjected to 4 h of H_2_O_2_-induced OSI. The JAK2 siRNA pretreatment significantly increased the cell viability (P<0.01, compared to H_2_O_2_ group) and partially reversed the cell shrinkage induced by the H_2_O_2_ treatment; the treatment with JAK2 siRNA alone had no effect on the OD value of the cells (Figure 4A, 4B and Table S4). As depicted in Figure 4C, the pretreatment with JAK2 siRNA produced a significant decrease in the cellular apoptotic index compared to the H_2_O_2_ group (P<0.01).

**Figure 5:**
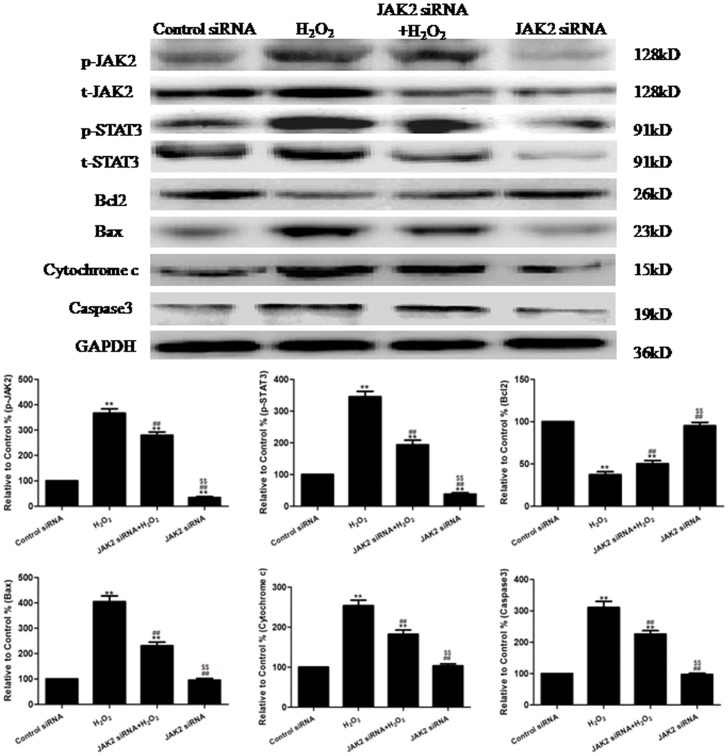
The effects of JAK2 siRNA on the levels of p-JAK2 and p-STAT3 and the expression of Bcl2, Caspase3, Bax and Cytochrome c in H_2_O_2_-injured HUVECs (treated for 4 h). Representative images of the Western blots are shown. The results are expressed as the mean ± SEM, n = 6, **P<0.01 compared to the Control siRNA group, ^##^P<0.01 compared to the H_2_O_2_ group, ^$$^P<0.01 compared to the JAK2 siRNA+H_2_O_2_ group.

As observed from the results of the Western blotting analysis (Figure 5), when the cells were treated with JAK2 siRNA+H_2_O_2_, a significant decrease in p-JAK2 and p-STAT3 and the expression of Caspase3, Bax and Cytochrome c was observed (P<0.01, compared to the H_2_O_2_ group), and a significant increase in Bcl2 expression was observed (P<0.01, compared to the H_2_O_2_ group). From the results of the immunofluorescence analysis (Figure S3A and S3B), when the cells were treated with JAK2 siRNA+H_2_O_2_, there was a significant decrease in p-JAK2 and p-STAT3 compared to the H_2_O_2_ group (P<0.01).

Compared to the control group, treatment with melatonin (125, 250 and 500 µM) for 2, 4 and 8 h did not have a significant influence on the cell viability or proliferation ability (Figure S4 and Table S5).

**Figure 6:**
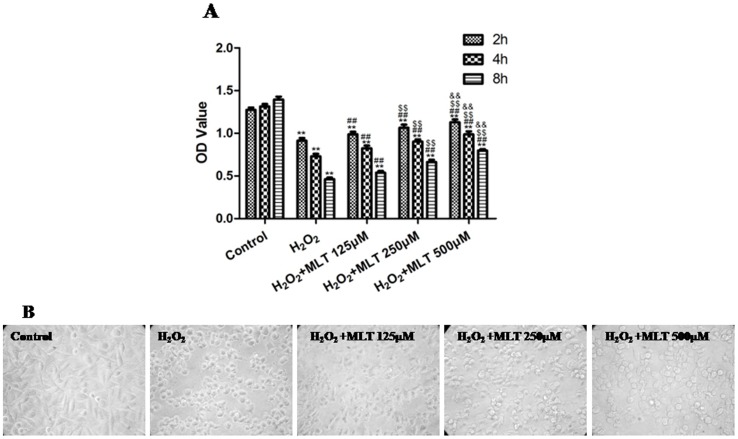
The effects of melatonin on the viability and morphology of H_2_O_2_-injured HUVECs (treated for 4 h). (A) The viability of the HUVECs was assessed by performing an MTT assay, and the viability was expressed as an OD value. (B) The cell morphology (treated for 4 h) was observed under an inverted/phase-contrast microscope, and images were obtained. Significant cell shrinkage and a decrease in the cellular attachment rate were observed in the H_2_O_2_ group. However, melatonin treatment reduced the H_2_O_2_-induced cell shrinkage and decreased the cellular attachment rate. The results are expressed as the mean ± SEM, n = 6, **P<0.01 compared to the control group, ^##^P<0.01 compared to the H_2_O_2_ group, ^$$^P<0.01 vs. the H_2_O_2_+MLT (125 µM) group, ^&&^P<0.01 compared to the H_2_O_2_+MLT (250 µM) group. MLT, melatonin. OD, optical density.

The HUVECs were subjected to 2, 4, and 8 h of H_2_O_2_ treatment in the absence or presence of melatonin (125, 250 and 500 µM), which significantly increased the cell viability compared to the respective H_2_O_2_ groups (P<0.01); the effects of 500 µM melatonin for 2, 4 and 8 h were the most significant compared to the other 2 concentrations (Figure 6A and Table S6). In addition, treatment with H_2_O_2_ plus melatonin (125, 250 and 500 µM) attenuated H_2_O_2_-induced cell shrinkage and improved the attachment rate of the cells (Figure 6B). Based on these results, treatment with 500 µM melatonin and 400 µM H_2_O_2_ for 4 h was selected for the further experiments.

**Figure 7:**
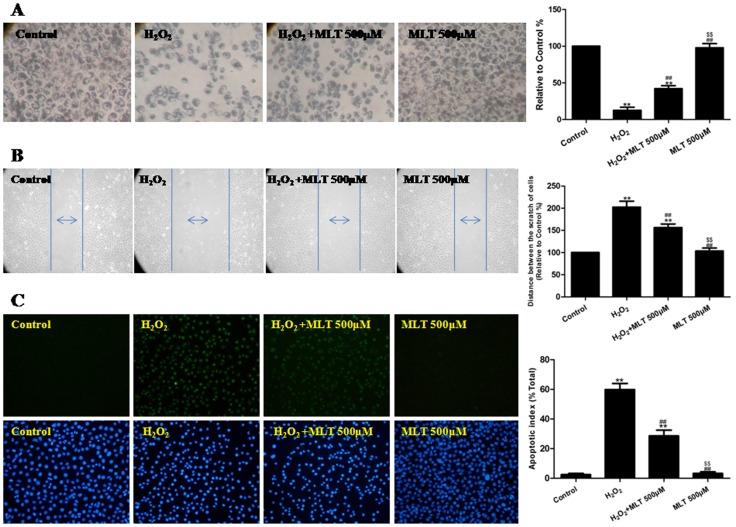
The effects of melatonin on the adhesive ability, migratory ability and apoptotic rate of H_2_O_2_-injured HUVECs (treated for 4 h). (A) The adhesive ability of the HUVECs was assessed by performing an adhesion assay, and cell adhesion was expressed as an adhesion ratio. The number of adherent cells in the control group was set at 100%. (B) The migratory ability of the HUVECs was assessed by performing a wound healing assay, and the migratory ability was expressed as the mean distance between the two ends of the scratch. The mean distance in the control group was set at 100%. (C) The apoptosis of the HUVECs was assessed by performing a TUNEL assay, and cellular apoptosis was expressed as the apoptotic index. TUNEL staining was performed to stain the nuclei of the apoptotic cells (green), and DAPI was used to stain all of the nuclei (blue). The apoptotic index was expressed as the number of positively stained apoptotic cells/the total number of cells counted × 100%. The results are expressed as the mean ± SEM, n = 6, ^**^P<0.01 compared to the control group, ^##^P<0.01 compared to the H_2_O_2_ group. MLT, melatonin. OD, optical density.

As demonstrated in Figure 7A, the cell adhesive ratio decreased significantly after incubation with H_2_O_2_ (P<0.01, compared to the control group), and the melatonin (500 µM) treatment significantly increased the cell adhesive ratio (P<0.01, compared to the H_2_O_2_ group). As demonstrated in Figure 7B, the distance between the scratches increased significantly after treatment with H_2_O_2_ (P<0.01, compared to the control group), whereas the melatonin (500 µM) treatment significantly decreased the distance (P<0.01, compared to the H_2_O_2_ group). As demonstrated in Figure 7C, the cellular apoptotic index increased significantly after treatment with H_2_O_2_ (P<0.01, compared to the control group), and the melatonin (500 µM) treatment significantly decreased the cell apoptotic index (P<0.01, compared to the H_2_O_2_ group). Compared to the control group, MLT treatment alone had no effect on the adhesive ratio, distance between the scratches and cellular apoptotic index of cells (P>0.05).

**Figure 8:**
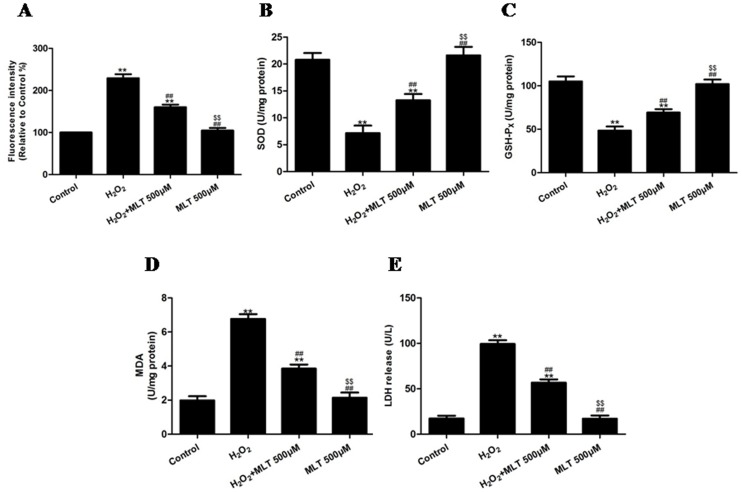
The effects of melatonin on the intracellular ROS, SOD, GSH-Px, and MDA levels and LDH release of H_2_O_2_-injured HUVECs (treated for 4 h). (A) The intracellular ROS level in the HUVECs was assessed by measuring the intensity of DCFH fluorescence, and cell viability was expressed as the fluorescence intensity. The fluorescence intensity in the control group was set at 100%. (B) The intracellular SOD level of HUVECs treated with H_2_O_2_ in the absence or presence of melatonin. (C) The intracellular GSH-Px level of HUVECs treated with H_2_O_2_ in the absence or presence of melatonin. (D) The intracellular MDA level of HUVECs treated with H_2_O_2_ in the absence or presence of melatonin. (E) The release of LDH from HUVECs treated with H_2_O_2_ in the absence or presence of melatonin. The results are expressed as the mean ± SEM, n = 6, **P<0.01 compared to the control group, ^##^P<0.01 compared to the H_2_O_2_ group. MLT, melatonin.

The intracellular ROS concentration was determined by measuring the intensity of DCFH fluorescence. When the DCFH-DA-labeled cells were incubated for 2 h, a sudden increase in the fluorescence intensity indicated the oxidation of DCFH-DA by intracellular radicals. As demonstrated in Figure 8A, the fluorescence intensity increased significantly after treatment with H_2_O_2_ (P<0.01, compared to the control group), whereas treatment with melatonin (500 µM) significantly decreased the fluorescence intensity (P<0.01, compared to the H_2_O_2_ group). Treating the cells with H_2_O_2_ for 4 h decreased the SOD and GSH-Px levels, respectively, (P<0.01, compared to the control group). However, incubation with melatonin (500 µM) significantly attenuated the changes in the content of SOD and GSH-Px (Figure 8B and 8C, respectively) (P<0.01, compared to the H_2_O_2_ group). In addition, H_2_O_2_ treatment for 4 h increased the intracellular MDA and LDH release, respectively (P<0.01, compared to the control group), however incubation with melatonin (500 µM) produced a marked decrease in the intracellular level of MDA and LDH (P<0.01, compared to the H_2_O_2_ group, Figure 8D and 8E, respectively). Compared to the control group, MLT treatment alone had no effect on the ROS, SOD, GSH-Px, MDA and LDH of cells (P>0.05).

**Figure 9:**
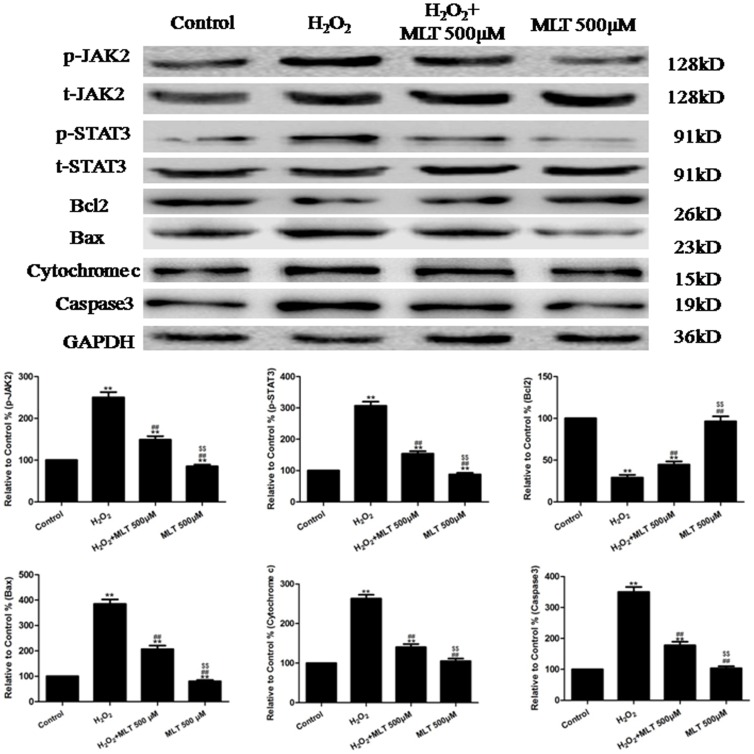
The effects of melatonin on p-JAK2 and p-STAT3 and the expression of Bcl2, Caspase3, Bax and Cytochrome c in H_2_O_2_-injured HUVECs (treated for 4 h). Representative images of the Western blots are shown. The results are expressed as the mean ± SEM, n = 6, **P<0.01 compared to the control group, ^##^P<0.01 compared to the H_2_O_2_ group. MLT, melatonin.

As demonstrated in the results of the Western blotting analysis (Figure 9), when the cells were treated with H_2_O_2_+ melatonin (500 µM), the levels of p-JAK2 and p-STAT3 and the expression of Caspase3, Bax and Cytochrome c decreased significantly (compared to the H_2_O_2_ group, P<0.01), whereas the expression of Bcl2 increased significantly (compared to the H_2_O_2_ group, P<0.01). Compared to the control group, MLT treatment alone slightly decreased the p-JAK2 and p-STAT3 expression of cells (P<0.01). As demonstrated in the results of the immunofluorescence analysis (Figure S4A and S4B), the levels of p-JAK2 and p-STAT3 decreased significantly when the cells were treated with H_2_O_2_+ melatonin (500 µM) (compared to the H_2_O_2_ group, P<0.01).

## Discussion

JAK2/STAT3 has been convincingly implicated in EC-fate determination during vasculogenesis and angiogenesis [33]. For example, the JAK2/STAT3 signaling pathway plays an important role in the angiogenesis of non-small cell lung cancer (NSCLC), and blocking this pathway may inhibit the expression of angiogenic cytokines. The JAK2/STAT3 signaling pathway may be a critical therapeutic target for the treatment of angiogenesis in NSCLC [34]. Dong Y and colleagues provided the first evidence that cucurbitacin E (CuE) inhibited tumor angiogenesis by inhibiting the vascular endothelial growth factor receptor 2 (VEGFR2)-mediated JAK2/STAT3 and mitogen-activated protein kinase (MAPK) signaling pathways, and CuE might be a potential candidate in angiogenesis-related disease therapy [35]. Choi JS and colleagues suggested that quercetin and rutin inhibit Cu^2+^-oxidized low-density lipoprotein-induced endothelial apoptosis by modulating the JAK2/STAT3 pathway and that rutin might modulate a signaling crosstalk between the JAK2 and MAPK pathways [36]. In addition, the therapeutic effect of recombinant human erythropoietin (rhEPO) on the subsequent vasospasm after subarachnoid hemorrhage may relate to its inhibition of endothelial apoptosis in cerebral arteries, which may be mediated in part by the JAK2/STAT3 signaling pathway [37]. In summary, the JAK2/STAT3 signaling pathway plays an essential role in regulating proliferation, differentiation and apoptosis in both embryonic and adult ECs.

The JAK2/STAT3 signaling pathway is also involved in the process of oxidative stress-induced injury. In the research of vascular smooth muscle cells (VSMCs), Li J and colleagues found that the inhibition of JAK2/STAT3 could significantly attenuate H_2_O_2_-induced apoptosis and block the H_2_O_2_-induced activation of STAT3, and their data indicated that leukocyte antigen-related protein regulates the Fyn/JAK2/STAT3 and Fyn/p38 MAPK pathways, which are involved in ROS-induced apoptosis [38]. Kim US and colleagues confirmed the H_2_O_2_-induced phosphorylation of JAK2 and STAT3 in lens epithelial cells (LECs): the phosphorylation of JAK2 and STAT3 was suppressed in cells pretreated with AG490, whereas AG490 notably enhanced cell survival and decreased cell necrosis [39]. In addition, the inhibition of JAK2/STAT3 signaling could also reduce H_2_O_2_-induced OSI in neuroglial cells [40], astrocytes [41], aortic endothelial cells [42] and proximal tubule cells [43]. From our studies, we found that AG490 and JAK2 siRNA inhibited OSI, as evidenced by improved cell viability and a decreased apoptotic index. As expected, AG490 and JAK2 siRNA effectively inhibited p-JAK2 and p-STAT3. These results demonstrate that the inhibition of the JAK2/STAT3 signaling pathway provides a protective effect against endothelial OSI.

Melatonin has potent antioxidant properties that may prevent the development of atherosclerosis and other consequences of aging [44]. In addition, the direct antioxidant activity of melatonin and its stimulatory effect on antioxidant enzyme activities may have clinical implications for the treatment of hyperlipidemia [45] in which the increased production of free radicals would be expected. The detailed mechanisms underlying melatonin’s protective effects have varied extensively among studies. Nuclear factor-kappaB [46], p38 mitogen-activated protein kinase, c-Jun N-terminal kinase (JNK) [47], Sirtuin 1 [48], hemeoxygenase-1 [49], eNOS [50], PI3K/Akt [51], autophagy [52] and the JAK2/STAT3 signaling pathway were reported to play a role in the protective effects of melatonin in EC oxidative stress injury. However, other evidence has demonstrated that melatonin receptor/Gα(16) coupling was capable of triggering the production of cytokines, including IL-6, and that this autocrine loop might account for the subsequent STAT3 phosphorylation at Tyr(705) [27]. By increasing STAT3 phosphorylation, melatonin might be an effective cytoprotective agent against palmitic acid-based cytotoxicity through the modulation of cell survival and inflammatory responses in astroglial cells [28]. Of note, melatonin can protect the liver against the I/R injury associated with the inhibition of JAK/STAT signaling in a rat hepatic ischemia/reperfusion injury model [53]. Above all, we speculated that the JAK2/STAT3 signaling pathway may play a regulatory role in the biological effects of melatonin. From our studies, we confirmed that melatonin conferred protection to HUVECs against H_2_O_2_, which was evidenced by the improved cell viability, adhesive ability, and migratory ability and decreased apoptotic index.

Mitochondria initiate two distinct apoptotic pathways, the intrinsic mitochondrial pathway and the extrinsic membrane death receptor pathway. A majority of the anti-OSI drugs prevent apoptosis by regulating the intrinsic mitochondrial pathway [54], [55]. Bax, Bak, Cytochrome c, and Caspase3 play important roles in the OSI-induced apoptotic process, and are all important members of the intrinsic mitochondrial pathway [54], [55]. Studies have demonstrated that the regulation of JAK2/STAT3 signaling by diverse drugs can induce apoptosis through the intrinsic mitochondrial pathway. For example, Duw and colleagues. illustrated the biological significance of JAK2/STAT3 signaling for colorectal cancer apoptosis and provided novel evidence that the inhibition of JAK2/STAT3 induced apoptosis via the mitochondrial apoptotic pathway [56]. It has also been reported that an adenovirus-vector carrying basic fibroblast growth factor siRNA reduced STAT3 phosphorylation and ultimately resulted in the collapse of the mitochondrial membrane potential and the induction of mitochondrial-related apoptosis in U251 glioma cells [57]. During the process of apoptosis, mitochondria serve as a source of ROS, which is generated by the reduction of the mitochondrial membrane potential, and the enhanced ROS production is related to the apoptotic response induced by OSI [58]. Lipid peroxidation is one of the primary events in cell OSI [32], and MDA is a by-product of the lipid peroxidation induced by excessive ROS and is widely used as a biomarker of oxidative stress. However, cells are equipped with several antioxidants for the prevention of free-radical damage: SOD and GSH-Px, along with other enzymatic and non-enzymatic antioxidants, play pivotal roles in preventing the cellular damage caused by ROS. Therefore, intracellular ROS can be effectively eliminated by the combined action of SOD, GSH-Px and other endogenous antioxidants, providing a repair mechanism for oxidized membrane components [32]. In the present study, significant decreases in SOD and GSH-Px were observed in HUVECs after the exposure to H_2_O_2_, indicating the impairment of antioxidant defenses. In addition, an obvious elevation of MDA production was associated with an increase in LDH release. Nonetheless, when HUVECs were co-treated with melatonin, these H_2_O_2_-induced cellular events were blocked to a great extent. Importantly, in addition to the down-regulation of H_2_O_2_-induced JAK2/STAT3 signaling, the melatonin treatment also down-regulated H_2_O_2_-induced mitochondrial apoptotic pathway-related proteins (Bax, Bak, Cytochrome c, and Caspase3). These results suggest that the enhancement of endogenous antioxidant preservation and attenuation the mitochondrial apoptotic pathway may represent a major mechanism of cellular protection by melatonin.

In summary, our study documents that the inhibition of the JAK2/STAT3 signaling pathway results in a protective effect against endothelial OSI and that JAK2/STAT3 signaling is a crucial link in endothelial OSI. In addition, melatonin attenuates endothelial OSI by inhibiting the JAK2/STAT3 signaling pathway.

## Supporting Information

Figure S1 **The effects of low concentration of H_2_O_2_ on HUVEC viability and the levels of p-JAK2 and p-STAT3.** (A) The viability of the HUVECs was assessed by performing an MTT assay, and the viability was expressed as an OD value. (B) Representative images of the Western blots are shown (treated for 4 h). The results are expressed as the mean ± SEM, n = 6, ^**^P<0.01 compared to the control group, ^##^P<0.01 compared to the 400 µM H_2_O_2_ group, ^$$^P<0.01 compared to the 50 µM H_2_O_2_ group. OD, optical density.
(TIF)

Figure S2 **AG490 on the levels of p-JAK2 and p-STAT3 in H_2_O_2_-injured HUVECs (treated for 4 h).** (A) Representative images of the p-JAK2 immunofluorescence are shown. (B) Representative images of the p-STAT3 immunofluorescence are shown. The results are expressed as the mean ± SEM, n = 6, ^**^P<0.01 compared to the control group, ^##^P<0.01 compared to the H_2_O_2_ group, ^$$^P<0.01 compared to the H_2_O_2_+ AG490 (20 µM) group.
(TIF)

Figure S3 **The effects of JAK2 siRNA on the levels of p-JAK2 and p-STAT3 in H_2_O_2_-injured HUVECs (treated for 4 h).** (A) Representative images of the p-JAK2 immunofluorescence are shown. (B) Representative images of the p-STAT3 immunofluorescence are shown. The results are expressed as the mean ± SEM, n = 6, ^**^P<0.01 compared to the Control siRNA group, ^##^P<0.01 compared to the H_2_O_2_ group, ^$$^P<0.01 compared to the JAK2 siRNA+H_2_O_2_ group.
(TIF)

Figure S4 **The effects of melatonin on the viability of normal HUVECs.** The viability of the HUVECs was assessed by performing an MTT assay, and the viability was expressed as an OD value. The results are expressed as the mean ± SEM, n = 6. MLT, melatonin.
(TIF)

Figure S5 **The effects of melatonin on the levels of p-JAK2 and p-STAT3 in H_2_O_2_-injured HUVECs (treated for 4 h).** (A) Representative images of the p-JAK2 immunofluorescence are shown. (B) Representative images of the p-STAT3 immunofluorescence are shown. The results are expressed as the mean ± SEM, n = 6, ^**^P<0.01 compared to the control group, ^##^P<0.01 compared to the H_2_O_2_ group. ^$$^P<0.01 compared to the H_2_O_2_+MLT 500 µM group. MLT, melatonin.
(TIF)

Table S1 **The effects of H_2_O_2_ on HUVEC viability.** The viability of the HUVECs was assessed by performing an MTT assay, and the viability was expressed as an OD value. The results are expressed as the mean ± SEM, n = 6, ^**^P<0.01 compared to the control group, ^##^P<0.01 compared to the 100 µM H_2_O_2_ group, ^$$^P<0.01 compared to the 200 µM H_2_O_2_ group. OD, optical density.
(DOCX)

Table S2 **The effects of low concentration of H_2_O_2_ on HUVEC viability.** The viability of the HUVECs was assessed by performing an MTT assay, and the viability was expressed as an OD value. The results are expressed as the mean ± SEM, n = 6, ^**^P<0.01 compared to the control group, ^##^P<0.01 compared to the 400 µM H_2_O_2_ group, ^$$^P<0.01 compared to the 50 µM H_2_O_2_ group. OD, optical density.
(DOCX)

Table S3 **The effects of AG490 on the viability of H_2_O_2_-injured HUVECs (treated for 4 h).** The viability of the HUVECs was assessed by performing an MTT assay, and the viability was expressed as an OD value. The results are expressed as the mean ± SEM, n = 6, ^**^P<0.01 compared to the control group, ^##^P<0.01 compared to the H_2_O_2_ group, ^$$^P<0.01 compared to the H_2_O_2_+ AG (20 µM) group. AG, AG490; OD, optical density.
(DOCX)

Table S4 **The effects of JAK2 siRNA on the viability of H_2_O_2_-injured HUVECs (treated for 4 h).** (A) The viability of the HUVECs was assessed by performing an MTT assay, and the viability was expressed as an OD value. The results are expressed as the mean ± SEM, n = 6, ^**^P<0.01 compared to the Control siRNA group, ^##^P<0.01 compared to the H_2_O_2_ group, ^$$^P<0.01 compared to the JAK2 siRNA+H_2_O_2_ group. OD, optical density.
(DOCX)

Table S5 **The effects of melatonin on the viability of normal HUVECs.** The viability of the HUVECs was assessed by performing an MTT assay, and the viability was expressed as an OD value. The results are expressed as the mean ± SEM, n = 6. MLT, melatonin; OD, optical density.
(DOCX)

Table S6 **The effects of melatonin on the viability of H_2_O_2_-injured HUVECs (treated for 4 h).** The viability of the HUVECs was assessed by performing an MTT assay, and the viability was expressed as an OD value. The results are expressed as the mean ± SEM, n = 6, ^**^P<0.01 compared to the control group, ^##^P<0.01 compared to the H_2_O_2_ group, ^$$^P<0.01 vs. the H_2_O_2_+MLT (125 µM) group, ^&&^P<0.01 compared to the H_2_O_2_+MLT (250 µM) group. MLT, melatonin. OD, optical density.
(DOCX)
