# Supplementary figures and images for: New Role of JAK2/STAT3 Signaling in Endothelial Cell Oxidative Stress Injury and Protective Effect of Melatonin
Source: PLoS One. 2013 Mar 6;8(3):e57941. doi: 10.1371/journal.pone.0057941 (PMC3590213; doi:10.1371/journal.pone.0057941)

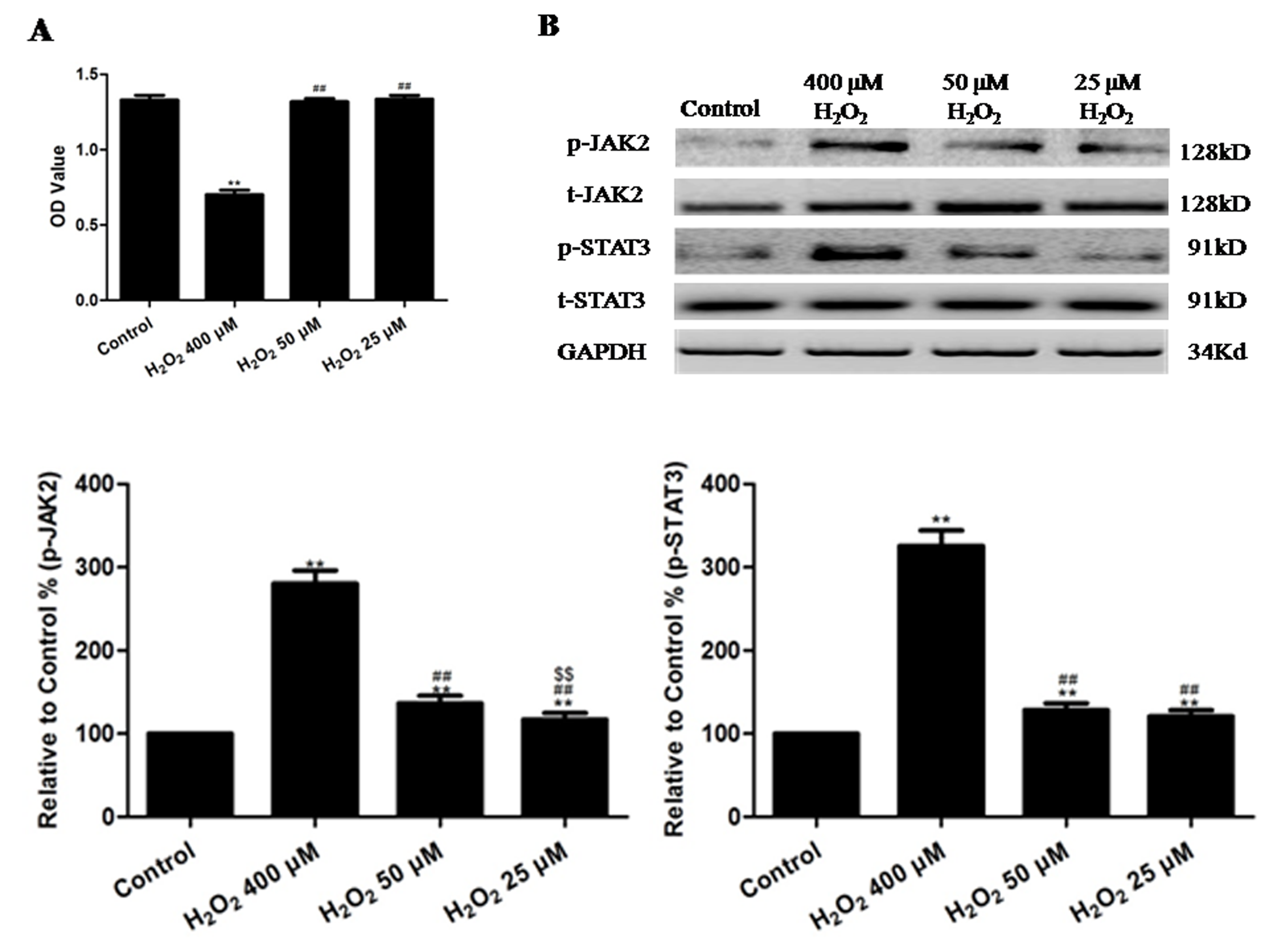

Supplement: Figure S1 — The effects of low concentration of H2O2 on HUVEC viability and the levels of p-JAK2 and p-STAT3. (A) The viability of the HUVECs was assessed by performing an MTT assay, and the viability was expressed as an OD value. (B) Representative images of the Western blots are shown (treated for 4 h). The results are expressed as the mean ± SEM, n = 6, **P<0.01 compared to the control group, ##P<0.01 compared to the 400 µM H2O2 group, $$P<0.01 compared to the 50 µM H2O2 group. OD, optical density. (TIF) [file pone.0057941.s001.tif]

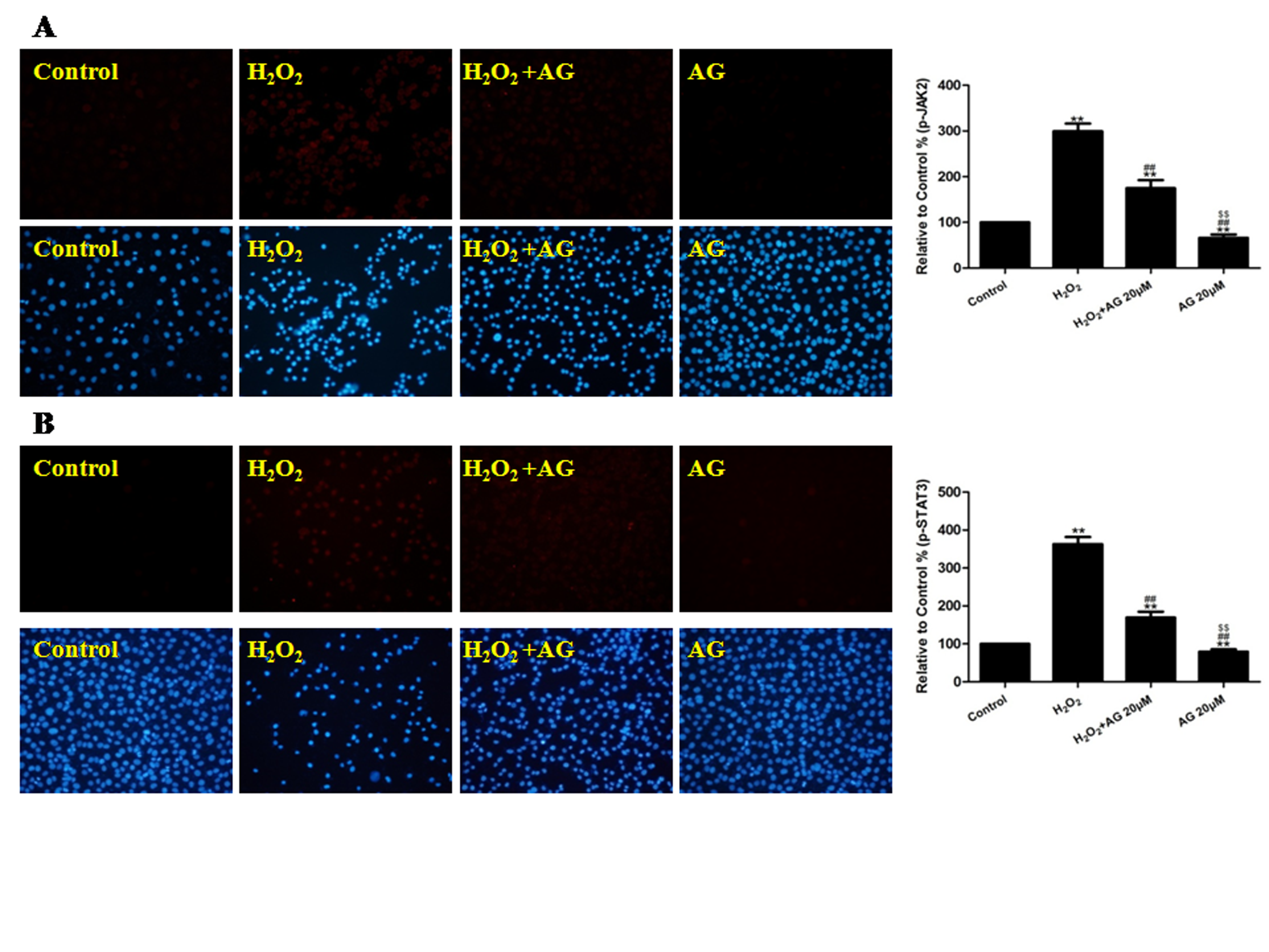

Supplement: Figure S2 — AG490 on the levels of p-JAK2 and p-STAT3 in H2O2-injured HUVECs (treated for 4 h). (A) Representative images of the p-JAK2 immunofluorescence are shown. (B) Representative images of the p-STAT3 immunofluorescence are shown. The results are expressed as the mean ± SEM, n = 6, **P<0.01 compared to the control group, ##P<0.01 compared to the H2O2 group, $$P<0.01 compared to the H2O2+ AG490 (20 µM) group. (TIF) [file pone.0057941.s002.tif]

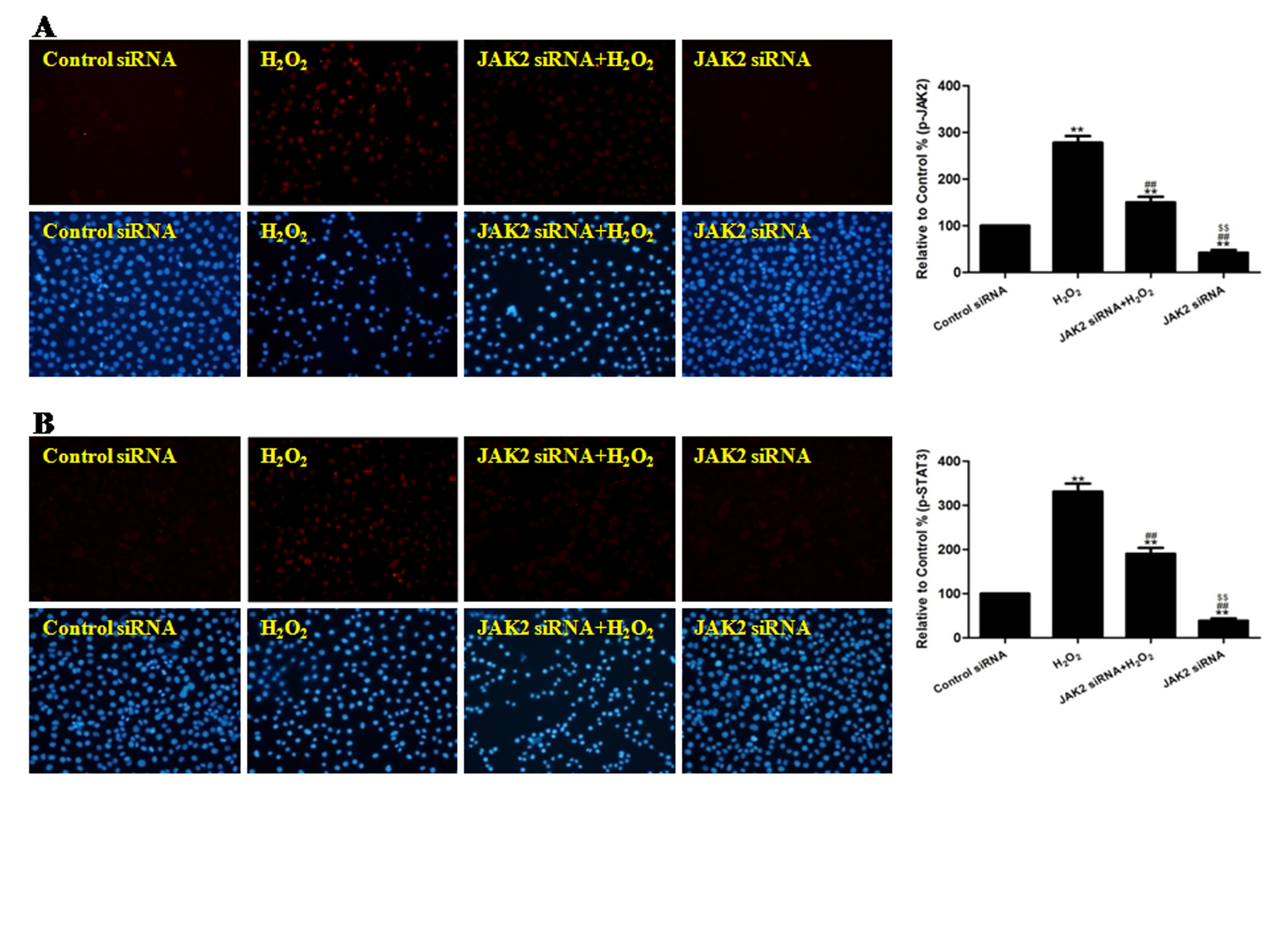

Supplement: Figure S3 — The effects of JAK2 siRNA on the levels of p-JAK2 and p-STAT3 in H2O2-injured HUVECs (treated for 4 h). (A) Representative images of the p-JAK2 immunofluorescence are shown. (B) Representative images of the p-STAT3 immunofluorescence are shown. The results are expressed as the mean ± SEM, n = 6, **P<0.01 compared to the Control siRNA group, ##P<0.01 compared to the H2O2 group, $$P<0.01 compared to the JAK2 siRNA+H2O2 group. (TIF) [file pone.0057941.s003.tif]

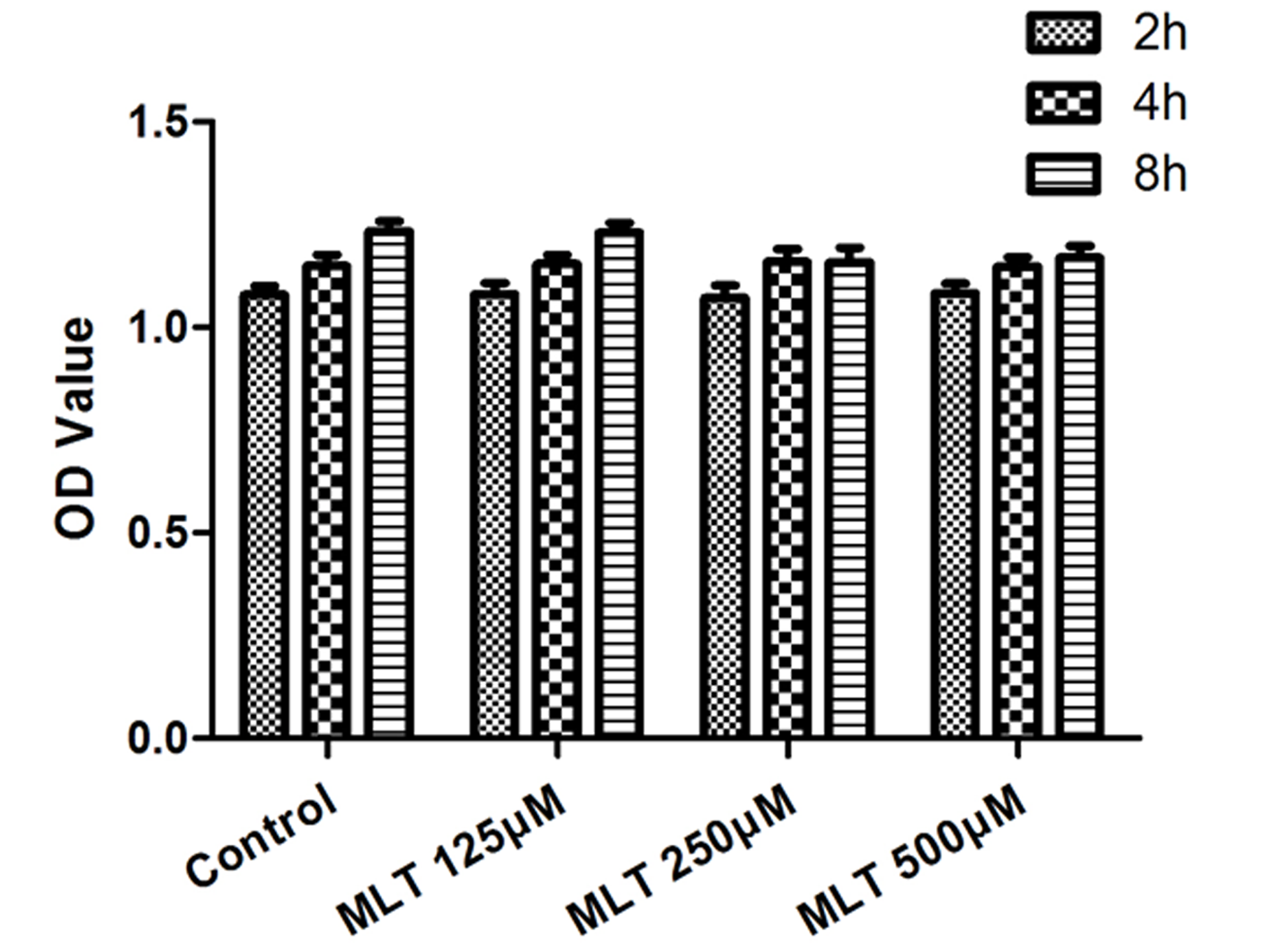

Supplement: Figure S4 — The effects of melatonin on the viability of normal HUVECs. The viability of the HUVECs was assessed by performing an MTT assay, and the viability was expressed as an OD value. The results are expressed as the mean ± SEM, n = 6. MLT, melatonin. (TIF) [file pone.0057941.s004.tif]

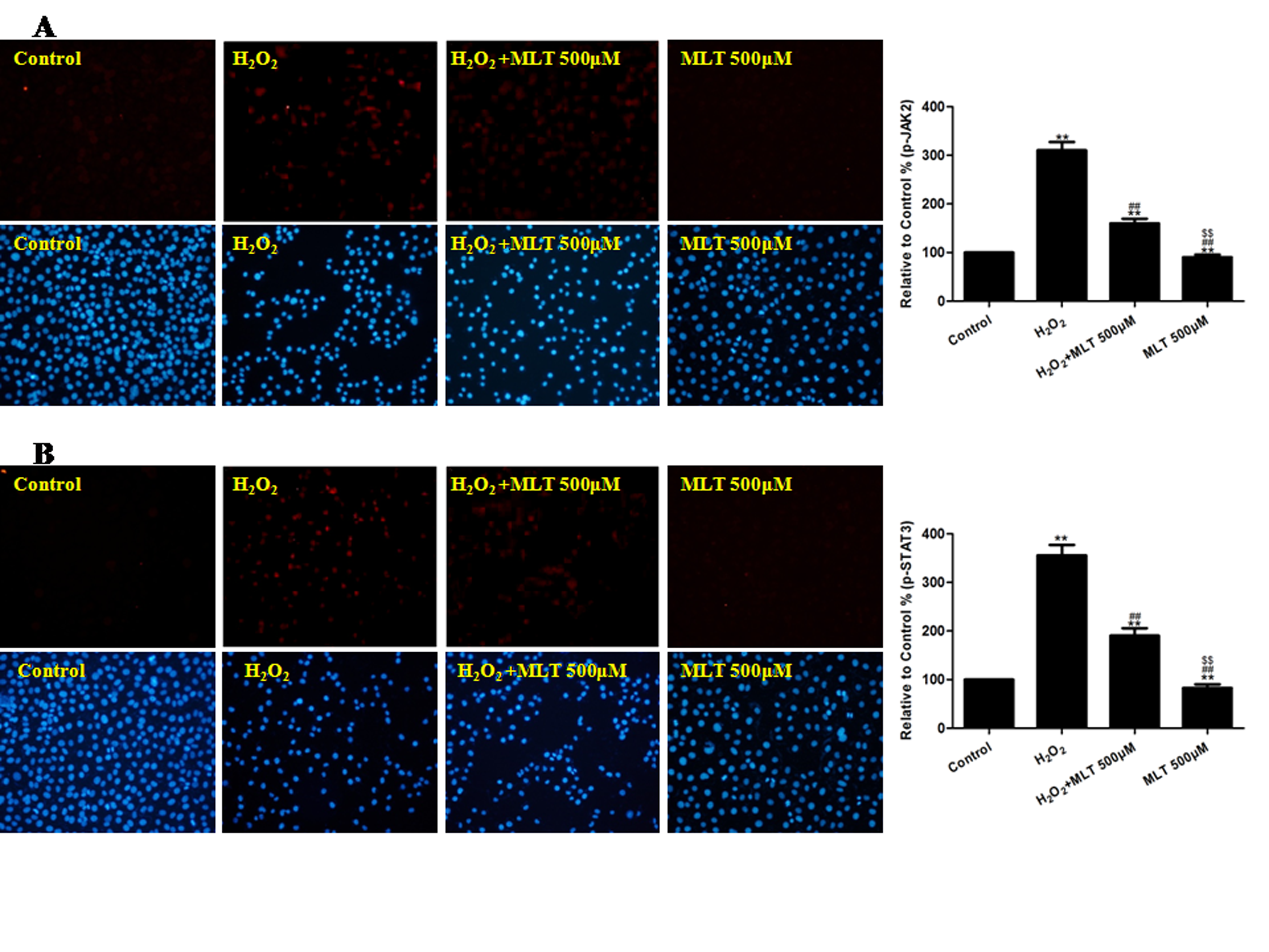

Supplement: Figure S5 — The effects of melatonin on the levels of p-JAK2 and p-STAT3 in H2O2-injured HUVECs (treated for 4 h). (A) Representative images of the p-JAK2 immunofluorescence are shown. (B) Representative images of the p-STAT3 immunofluorescence are shown. The results are expressed as the mean ± SEM, n = 6, **P<0.01 compared to the control group, ##P<0.01 compared to the H2O2 group. $$P<0.01 compared to the H2O2+MLT 500 µM group. MLT, melatonin. (TIF) [file pone.0057941.s005.tif]
