# Supplementary material for: New Role of JAK2/STAT3 Signaling in Endothelial Cell Oxidative Stress Injury and Protective Effect of Melatonin
Source: PLoS One. 2013 Mar 6;8(3):e57941. doi: 10.1371/journal.pone.0057941 (PMC3590213; doi:10.1371/journal.pone.0057941)
Supplement: Table S1 — The effects of H2O2 on HUVEC viability. The viability of the HUVECs was assessed by performing an MTT assay, and the viability was expressed as an OD value. The results are expressed as the mean ± SEM, n = 6, **P<0.01 compared to the control group, ##P<0.01 compared to the 100 µM H2O2 group, $$P<0.01 compared to the 200 µM H2O2 group. OD, optical density. (DOCX) [file pone.0057941.s006.docx]

**Supplement Table 1 The effects of** **H_2_O_2_ on HUVEC viability**

|  | Control | 100 μM H_2_O_2_ | 200 μM H_2_O_2_ | 400 μM H_2_O_2_ |
| --- | --- | --- | --- | --- |
| 2h | 1.236±0.036 | 1.120±0.026^**^ | 0.954±0.022^**##^ | 0.811±0.027^**##$$^ |
| 4h  8h | 1.245±0.029  1.340±0.037 | 0.971±0.031^**^  0.847±0.024^**^ | 0.872±0.030^**##^  0.693±0.026^**##^ | 0.672±0.025^**##$$^  0.513±0.022^**##$$^ |
