# Supplementary material for: New Role of JAK2/STAT3 Signaling in Endothelial Cell Oxidative Stress Injury and Protective Effect of Melatonin
Source: PLoS One. 2013 Mar 6;8(3):e57941. doi: 10.1371/journal.pone.0057941 (PMC3590213; doi:10.1371/journal.pone.0057941)
Supplement: Table S2 — The effects of low concentration of H2O2 on HUVEC viability. The viability of the HUVECs was assessed by performing an MTT assay, and the viability was expressed as an OD value. The results are expressed as the mean ± SEM, n = 6, **P<0.01 compared to the control group, ##P<0.01 compared to the 400 µM H2O2 group, $$P<0.01 compared to the 50 µM H2O2 group. OD, optical density. (DOCX) [file pone.0057941.s007.docx]

**Supplement Table 2 The effects of low concentrations of H_2_O_2_ on HUVECs viability**

|  | Control | H_2_O_2_ 400μM | H_2_O_2_ 50μM | H_2_O_2_ 25μM | |  |
| --- | --- | --- | --- | --- | --- | --- |
| 4h | 1.329±0.0322 | 0.701±0.030^**^ | 1.318±0.022^##^ | | 1.335±0.025^##$$^ | |
