# Supplementary material for: New Role of JAK2/STAT3 Signaling in Endothelial Cell Oxidative Stress Injury and Protective Effect of Melatonin
Source: PLoS One. 2013 Mar 6;8(3):e57941. doi: 10.1371/journal.pone.0057941 (PMC3590213; doi:10.1371/journal.pone.0057941)
Supplement: Table S3 — The effects of AG490 on the viability of H2O2-injured HUVECs (treated for 4 h). The viability of the HUVECs was assessed by performing an MTT assay, and the viability was expressed as an OD value. The results are expressed as the mean ± SEM, n = 6, **P<0.01 compared to the control group, ##P<0.01 compared to the H2O2 group, $$P<0.01 compared to the H2O2+ AG (20 µM) group. AG, AG490; OD, optical density. (DOCX) [file pone.0057941.s008.docx]

**Supplement Table 3 The effects of AG490 on the viability of H_2_O_2_-injured HUVECs**

|  | Control | H_2_O_2_ | H_2_O_2_+AG | AG |
| --- | --- | --- | --- | --- |
| 4h | 1.221±0.0332 | 0.685±0.030^**^ | 0.822±0.022^**##^ | 1.217±0.025^##$$^ |
