# Supplementary material for: New Role of JAK2/STAT3 Signaling in Endothelial Cell Oxidative Stress Injury and Protective Effect of Melatonin
Source: PLoS One. 2013 Mar 6;8(3):e57941. doi: 10.1371/journal.pone.0057941 (PMC3590213; doi:10.1371/journal.pone.0057941)
Supplement: Table S4 — The effects of JAK2 siRNA on the viability of H2O2-injured HUVECs (treated for 4 h). (A) The viability of the HUVECs was assessed by performing an MTT assay, and the viability was expressed as an OD value. The results are expressed as the mean ± SEM, n = 6, **P<0.01 compared to the Control siRNA group, ##P<0.01 compared to the H2O2 group, $$P<0.01 compared to the JAK2 siRNA+H2O2 group. OD, optical density. (DOCX) [file pone.0057941.s009.docx]

**Supplement Table 4 The effects of JAK2 siRNA on the viability of H_2_O_2_-injured HUVECs**

|  | Control siRNA | H_2_O_2_ | JAK2 siRNA+H_2_O_2_ | JAK2 siRNA | |  |
| --- | --- | --- | --- | --- | --- | --- |
| 4h | 1.284±0.0382 | 0.702±0.031^**^ | 0.853±0.028^**##^ | | 1.259±0.040^##$$^ | |
