# Supplementary material for: New Role of JAK2/STAT3 Signaling in Endothelial Cell Oxidative Stress Injury and Protective Effect of Melatonin
Source: PLoS One. 2013 Mar 6;8(3):e57941. doi: 10.1371/journal.pone.0057941 (PMC3590213; doi:10.1371/journal.pone.0057941)
Supplement: Table S5 — The effects of melatonin on the viability of normal HUVECs. The viability of the HUVECs was assessed by performing an MTT assay, and the viability was expressed as an OD value. The results are expressed as the mean ± SEM, n = 6. MLT, melatonin; OD, optical density. (DOCX) [file pone.0057941.s010.docx]

**Supplement Table 5 The effects of melatonin on the viability of normal HUVECs**

|  | Control | MLT 125μM | MLT 250μM | MLT 500μM |
| --- | --- | --- | --- | --- |
| 2h | 1.079±0.021 | 1.080±0.028 | 1.072±0.030 | 1.083±0.024 |
| 4h  8h | 1.150±0.027  1.234±0.024 | 1.155±0.022  1.230±0.025 | 1.159±0.032  1.158±0.037 | 1.148±0.023  1.169±0.029 |
