# Supplementary material for: New Role of JAK2/STAT3 Signaling in Endothelial Cell Oxidative Stress Injury and Protective Effect of Melatonin
Source: PLoS One. 2013 Mar 6;8(3):e57941. doi: 10.1371/journal.pone.0057941 (PMC3590213; doi:10.1371/journal.pone.0057941)
Supplement: Table S6 — The effects of melatonin on the viability of H2O2-injured HUVECs (treated for 4 h). The viability of the HUVECs was assessed by performing an MTT assay, and the viability was expressed as an OD value. The results are expressed as the mean ± SEM, n = 6, **P<0.01 compared to the control group, ##P<0.01 compared to the H2O2 group, $$P<0.01 vs. the H2O2+MLT (125 µM) group, &&P<0.01 compared to the H2O2+MLT (250 µM) group. MLT, melatonin. OD, optical density. (DOCX) [file pone.0057941.s011.docx]

**Supplement Table 6 The effects of MLT on the viability of H_2_O_2_-injured HUVECs**

|  | Control | H_2_O_2_ | H_2_O_2_+MLT 125μM | H_2_O_2_+MLT 250μM | H_2_O_2_+MLT 500μM |
| --- | --- | --- | --- | --- | --- |
| 2h | 1.074±0.026 | 0.513±0.031^**^ | 0.546±0.030^**##^ | 0.644±0.036^**##$$^ | 0.780±0.034^**##$$&&^ |
| 4h  8h | 1.154±0.029  1.196±0.033 | 0.310±0.030^**^  0.187±0.019^**^ | 0.424±0.032^**##^  0.277±0.021^**##^ | 0.493±0.026^**##$$^  0.332±0.027^**##$$^ | 0.534±0.037^**##$$&&^  0.397±0.018^**##$$&&^ |
